# Recent Incarceration and HIV Risk Among Women Who Use Heroin

**DOI:** 10.1001/jamanetworkopen.2024.54455

**Published:** 2025-01-13

**Authors:** Kaitlyn Atkins, D’Andre Walker, Kathryn Noon, Chijindu Nwakama, Alana Snyder, Adela Luswetula, Jessie Mbwambo, Samuel Likindikoki, Haneefa Saleem

**Affiliations:** 1 Bloomberg School of Public Health, Johns Hopkins University, Baltimore, Maryland; 2 School of Applied Sciences, The University of Mississippi, Oxford; 3 Muhimbili University of Health and Allied Sciences, Dar es Salaam, Tanzania

## Abstract

**Importance:**

Women who use heroin in sub-Saharan Africa face elevated HIV risk linked to structural vulnerability including frequent incarceration. However, little is known about the association between incarceration and drug use and HIV outcomes among women who use heroin in Africa.

**Objective:**

To estimate associations between incarceration and adverse HIV-related and drug use-related outcomes among women who used heroin.

**Design, Setting, and Participants:**

This cross-sectional study included participants from Dar es Salaam, Tanzania, who were recruited using respondent-driven sampling. Eligible participants were women who used heroin who were aged 18 years or older and reported past-month heroin use. Data were collected from November 2018 to February 2019 and analyzed from September 2023 to May 2024.

**Exposure:**

The exposure was recent incarceration, defined as self-report of being held in prison or jail in the past 6 months.

**Main Outcomes and Measures:**

Main outcomes were self-reported HIV testing in the past 6 months, self-reported HIV status, and lifetime nonfatal overdose. Associations between recent incarceration and outcomes were examined using modified Poisson regression with robust variance estimation.

**Results:**

This study included 195 women who used heroin (median [IQR] age, 33 [27-39] years); 119 women (61%) reported incarceration in the past 6 months. In bivariate analyses, incarceration was associated with transactional sex (111 of 119 [93.3%]), symptoms of anxiety (104 of 119 [87.4%]), physical violence victimization (83 of 118 [70.3%]), and stigma from family (eg, 99 of 119 women [83.2%] reported being treated differently) and health care clinicians (eg, 46 of 119 women [38.7%] reported receiving poor health care). In adjusted analyses, incarceration was associated with higher prevalence of sexual concurrency (101 of 119 [84.9%] vs 41 of 76 [54.0%]; aPR, 1.43; 95% CI, 1.16-1.78), stimulant use (26 of 119 [21.9%] vs 3 of 76 [4.0%]; aPR, 5.60; 95% CI, 1.63-19.28), and lifetime nonfatal overdose (51 of 119 [42.9%] vs 17 of 76 [22.4%]; aPR, 1.62; 95% CI, 1.01-2.61). Among women who used heroin living with HIV, incarceration was associated with stopping HIV care (9 of 27 [33.3%] vs 1 of 24 [4.2%]; aPR, 9.74; 95% CI, 1.22-77.22).

**Conclusions and Relevance:**

In this cross-sectional study of HIV-related outcomes among recently incarcerated women who used heroin in sub-Saharan Africa, behavioral and structural vulnerabilities associated with incarceration were identified, which may exacerbate HIV disparities. Elevated stimulant use among recently incarcerated women who used heroin is of particular concern, given associations with adverse HIV outcomes. In the context of highly criminalized drug use, interventions targeting policing practices may be effective at reducing incarceration-associated risks. Findings could inform development and evaluation of multilevel interventions to reduce service interruptions and ensure linkage to HIV and substance use services during incarceration and reentry.

## Key Points

**Question:**

Is recent incarceration associated with adverse HIV and drug use outcomes among women who used heroin in Tanzania?

**Findings:**

In this cross-sectional study of 195 women who used heroin in Dar es Salaam, Tanzania, recent incarceration was common and was associated with sexual concurrency, stimulant use, lifetime nonfatal overdose, and missing HIV care appointments.

**Meaning:**

These findings suggest that in the context of highly criminalized drug use, multilevel interventions and collaborations across sectors should address HIV and substance use risks during incarceration and reentry.

## Teaser

This cross-sectional study examines associations between incarceration and adverse HIV-related and drug use-related outcomes among women who used heroin in Tanzania.

## Introduction

Globally, incarceration disproportionately affects people who use drugs, with an estimated 35% of the global prison population incarcerated for drug-related offenses.^1^ Compared with other settings, there is limited research on the association between incarceration and drug use in Africa; however, an emerging body of evidence suggests high rates of incarceration among people who use drugs in sub-Saharan Africa.^2,3,4^ Across sub-Saharan Africa, incarceration is associated with HIV, with HIV prevalence among incarcerated persons nearly double the general population^5^ and recent data estimating HIV prevalence of 15.6% among incarcerated persons across east and southern Africa.^6^ Increased risk for HIV transmission in carceral settings is associated with syringe sharing, inconsistent availability of condoms, preexposure prophylaxis or postexposure prophylaxis, and sexual violence.^5^ Furthermore, for people who use drugs and are living with HIV, incarceration has been associated with reduced access to treatment, preventing viral load suppression.^5^

People who use drugs in carceral settings also face multiple vulnerabilities placing them at elevated risk for overdose, particularly in the postrelease period, and other poor health outcomes.^7,8^ Research from the US has shown a large proportion of postrelease overdose-related deaths involve polysubstance use (ie, use of multiple substances in the same interval of time or simultaneously).^9,10^ Limited evidence from sub-Saharan Africa suggests polysubstance use among marginalized populations in sub-Saharan Africa is increasingly common and may complicate both HIV and drug treatment outcomes.^11,12^

Broadly, Tanzania’s carceral system has been characterized by crowding, human rights violations, and insufficient medical care.^13^ Limited HIV services are available and include voluntary testing, treatment (typically via escort offsite), and peer-led testing and prevention programs, although condoms and lubricants are not provided.^14^ Substance use services are more limited and individuals who are incarcerated and require specialized care (including methadone) are typically escorted offsite. Women are a minority of incarcerated persons in Tanzania, and their health and psychosocial needs are often poorly addressed in carceral settings.^15^ Furthermore, women who are incarcerated disproportionately engage in both sex work and drug use—both are criminalized in Tanzania and associated with elevated HIV burden in this population.^16,17^ However, there is a paucity of data on the incarceration experiences or incarceration-associated vulnerabilities of women who use drugs, particularly in sub-Saharan Africa.

Among women who use drugs, women who used heroin may be particularly vulnerable in carceral settings given high criminalization of heroin use and disproportionately high HIV prevalence in this population—estimated at 25% to 41%.^16,18^ Compared with other substances, heroin use among women has been more closely linked to syringe sharing, transactional sex, and severe stigma, all of which amplify vulnerability to HIV.^16,18^ Identifying risks associated with incarceration among women who used heroin in sub-Saharan Africa is necessary to support the public health needs of this vulnerable yet understudied population. To fill this gap, we aimed to (1) characterize incarceration history among women who used heroin in Tanzania, (2) identify factors of recent incarceration, and (3) estimate associations between incarceration and adverse outcomes associated with HIV and drug use.

## Methods

We conducted a cross-sectional survey with women who used heroin in Dar es Salaam, Tanzania, from November 2018 to February 2019. Analysis and reporting followed the Strengthening the Reporting of Observational Studies in Epidemiology (STROBE) reporting guideline.^19^

### Ethical Considerations

This study received ethical approvals from institutional review boards at the Muhimbili University of Health and Allied Sciences and the Johns Hopkins School of Public Health, and the National Health Research Ethics Review Sub-Committee of the Tanzania National Institute for Medical Research. All participants provided oral informed consent.

### Study Design, Participants, and Procedures

Women were eligible to participate if they were aged 18 years or older, reported heroin use in the past 30 days, and lived in Dar es Salaam, Tanzania. We recruited participants using respondent-driven sampling procedures.^20^ Following enrollment, trained study staff administered a structured survey in Swahili. Participants received 10 000 Tanzania shillings (approximately USD $4.30) as reimbursement for transportation and participation costs, and 6000 Tanzania shillings for each of up to 3 eligible women they recruited for the study.

### Variables

#### Exposure

Incarceration history was assessed through self-report. We asked whether women had been held in jail or prison in the past 6 months (yes or no). We also measured lifetime arrest history (yes or no), number of arrests in the past 6 months, and reasons for arrest (using drugs, selling drugs, selling sex, theft, assault, loitering, or others).

#### Study Outcomes

We assessed outcomes hypothesized to be associated with recent incarceration, all via self-report. Our primary outcomes associated with HIV were HIV testing in the past 6 months (yes or no) and HIV status (living with HIV, HIV-negative, or unknown status). Secondary outcomes associated with HIV included sexual concurrency in the past month (1 partner or less or 2 or more partners), enrolling in HIV care among women living with HIV (yes or no), and stopping HIV care in the past 6 months among women living with HIV (yes or no). Our primary drug use outcome was lifetime nonfatal overdose (yes or no). Secondary drug use outcomes were polydrug use in the past 6 months (use of marijuana, crack, cocaine, valium, prescription painkillers, opioids, amphetamines, or other illicit drugs), concurrent stimulant use in the past 6 months (use of cocaine, crack, or amphetamines), and lifetime injection drug use (yes or no).

#### Covariates

Covariates included in all models were age in years, education (less than primary, primary, secondary, or higher), and duration of heroin use (measured in years and categorized as 1 year or less, 2 to 9 years, and 10 years or more). For models not specific to women living with HIV, we also adjusted for self-reported HIV status.

#### Other Characteristics

We also assessed relationship status (no relationship or partnered and living separately, married or partnered and living together, or divorced or separated), number of dependents, homelessness in the last 6 months (yes or no), and current living situation (alone, with partner, with relatives, with other people who use drugs, with friends, or homeless). We calculated total income in the past 30 days by summing reported income from employment, partners, family members, friends, and sex work. We measured lifetime history of transactional sex (yes or no) and past 30 days income from selling sex in Tanzanian shillings. We used 6 items to measure lifetime exposure to stigma in health care settings and from family because of drug use.^21^ We also measured self-reported past-year forced sex and past-year physical violence by an intimate partner. We measured anxiety symptoms using the Generalized Anxiety Disorder-7 (α = 0.89)^2^ and depressive symptoms using the Patient Health Questionnaire-9 (α = 0.84).^3^

### Statistical Analysis

We described the prevalence of recent incarceration and examined the demographic and psychosocial characteristics of recently incarcerated women who used heroin, using χ^2^ tests for categorical variables and Wilcoxon rank-sum tests for continuous variables. We used modified Poisson regression with robust variance estimation to calculate adjusted prevalence ratios (aPRs) and 95% CIs for the associations between recent incarceration and HIV and drug use outcomes. We did not apply respondent driven sampling (RDS) weights given our goal was not to estimate generalizable prevalence estimates, and given unweighted regression has been shown to outperform RDS-weighted regression for dichotomous outcomes.^22^

We used directed acyclic graphs to identify a sufficient set of confounders for adjustment; all models were adjusted for age, education, and duration of heroin use. We assessed multicollinearity using variance inflation factor (VIF) to ensure there was no multicollinearity between our measured variables (ie, all VIFs less than 3.0). Data were analyzed from September 2023 to May 2024. Statistical significance was defined as 2-sided α < .05 or 95% CI not including the value of 1.00. Data were analyzed using Stata version 18.0 (StataCorp).

Regarding missing data, of the 200 women who used heroin in the study, 5 (2.5%) were missing data on incarceration; analyses were restricted to the remaining 195 women who used heroin. Overall, 19 of 195 women (10%) were missing responses for HIV status and 3 of 54 women (6%) who use heroin and were living with HIV were missing responses for stopping HIV care. There was no missing data for other outcomes. We examined the correlation matrix between outcomes and other available data and did not identify an observable pattern in missingness; we therefore used complete case analysis (list-wise deletion) in all models.

## Results

### Sample Characteristics

The final analytic sample included 195 women who used heroin (median [IQR] age was 33 [27-39] years). Of the 195 women who used heroin, 130 (66.7%) had been arrested at least once in the past 6 months, and 119 (61.0%) had been incarcerated in the past 6 months. The most common reasons for arrest were using drugs (67 of 130 [51.5%]) and selling sex (40 of 130 [30.8%]).

Compared with women who used heroin with no recent incarceration history, women who used heroin who were recently incarcerated were more likely to have used heroin for 10 or more years (32 of 119 [26.9%]; *P* = .003) and to report homelessness in the past 6 months (51 of 118 [43.2]; *P* = .02) (Table 1). Women who use heroin with incarceration history were otherwise similar to other women who used heroin in terms of age, education, and relationships.

**Table 1.** Demographic Characteristics of Women Who Use Heroin in Tanzania, by Incarceration Status (N = 195)

Characteristic	No./No. (%) [95% CI]		*P* value
	Recently incarcerated (n = 119)	Not recently incarcerated (n = 76)	
Age, median (IQR), y	32 (28-38)	35 (26-40.5)	.15
Education level			
Less than primary	42/119 (35.3) [27.2-44.4]	18/76 (23.7) [15.3-34.7]	.08
Completed primary	65/119 (54.6) [45.5-63.4]	43/76 (56.6) [45.1-67.4]	
Some secondary or higher	12/119 (10.1) [5.8-17.0]	15/76 (19.7) [12.2-30.4]	
Combined income, last 30 d, median (IQR), $^a^	86.21 (43.10-198.28)	76.72 (29.09-128.23)	.07
Duration of heroin use, y			
≤1	24/119 (20.2) [13.8-28.4]	28/76 (36.8) [26.6-48.4]	.03
2-9	63/119 (52.9) [43.9-61.8]	35/76 (46.1) [35.1-57.4]	
≥10	32/119 (26.9) [19.6-35.6]	13/76 (17.1) [10.1-27.5]	
Current relationship status			
Not in relationship	40/119 (33.6) [25.6-42.6]	18/76 (23.7) [15.3-34.7]	.12
Have partner not living with	34/119 (28.6) [21.1-37.4]	16/76 (21.1) [13.2-31.8]	
Married or living with a partner	31/119 (26.1) [18.9-34.8]	28/76 (36.8) [26.6-48.4]	
Divorced, separated, or widowed	14/119 (11.8) [7.1-19.0]	14/76 (18.4) [11.1-28.9]	
No. of dependents, median (IQR)	1 (0-2)	1 (0-3)	.47
Current living situation			
Live alone	17/119 (14.3) [9.0-21.9]	17 (22.4) [14.3-33.3]	.09
Live with partner	27/119 (22.7) [16.0-31.2]	27 (35.5) [25.5-47.0]	
Live with other relatives	31/119 (26.1) [18.9-34.8]	19 (25.0) [16.4-36.1]	
Live with other people who use drugs	26/119 (21.8) [15.3-30.3]	8 (10.5) [5.3-19.9]	
Live with friends	2/119 (1.7) [0.4-6.6]	1 (1.3) [0.2-9.0]	
No home	16/119 (13.4) [8.4-20.9]	4 (5.3) [2.0-13.4]	
Homelessness, past 6 mo^b,c^	51/118 (43.2) [34.1-52.7]	18/76 (23.7) [14.7-34.8]	.02
History of transactional sex, lifetime^b^	111/119 (93.3) [87.2-97.1]	54/76 (71.1) [59.5-80.9]	<.001
Any income from sex, last 30 d^b^	92/119 (77.3) [68.7-84.5]	34/76 (44.7) [33.3-56.6]	<.001
Income from sex, last 30 d, median (IQR), $^a^	52.00 (6.50-129.00)	0 (0-52.00)	<.001
Methadone use, lifetime^b^	18/119 (15.1) [9.2-22.8]	10/76 (13.2) [6.5-22.9]	.70

^a^ Responses were given in Tanzanian shillings (TZS); USD equivalent calculated based on average exchange rate during the study period (2320 TZS to 1 USD).

^b^ Yes responses shown.

^c^ Missing homelessness: 1 (0.5%).

Recently incarcerated women who used heroin were more likely than those who had not been recently incarcerated to report lifetime history of transactional sex and income from sex in the last 30 days and reported higher earnings from sex in the last month. Compared with those not recently incarcerated, women who used heroin with recent incarceration history were more likely to report psychosocial and structural vulnerabilities (Figure). Among women who used heroin and reported recent incarceration, 104 of 119 women (87.4%) reported moderate or severe anxiety symptoms compared with 49 of 76 women who used heroin but were not recently incarcerated (64.5%) (*P* < .001). Violence was also more common among women who used heroin and were recently incarcerated, with 83 of 118 recently incarcerated women (70.3%) reporting past-year physical violence vs 36 of 76 women who were not recently incarcerated (47.3%) (*P* = .02), and 50 of 115 women (43.5%) reported past-year sexual violence vs 19 of 73 women who were not recently incarcerated (26.0%) (*P* = .02). Women who were recently incarcerated and used heroin reported health care stigma at nearly twice the rate of other women who used heroin but were not recently incarcerated: 46 of 119 women (38.7%) reported receiving poor health care vs 14 of 76 women who were not recently incarcerated (18.4%) (*P* = .008); 38 of 119 women (31.9%) reported health care clinicians thought they were trying to get drugs from the clinic vs 11 of 76 women who were not recently incarcerated (14.5%) (*P* = .01); and 49 of 119 women (41.2%) reported not feeling listened to in health care vs 14 of 76 women who were not recently incarcerated (18.4%) (*P* = .01). Family stigma was also higher among women who were recently incarcerated; 99 of 119 women (83.2%) reported being treated differently because of drug use vs 45 of 76 women who were not recently incarcerated (59.2%) (*P* = .008), 100 of 119 women (84.0%) reported family looking down on them because of drug use vs 45 of 76 women who were not recently incarcerated (59.2%) (*P* = .002), and 99 of 119 women (83.2%) reported family seeing them as untrustworthy vs 43 of 76 women who were not recently incarcerated (56.6%) (*P* = .002).

**Figure.**
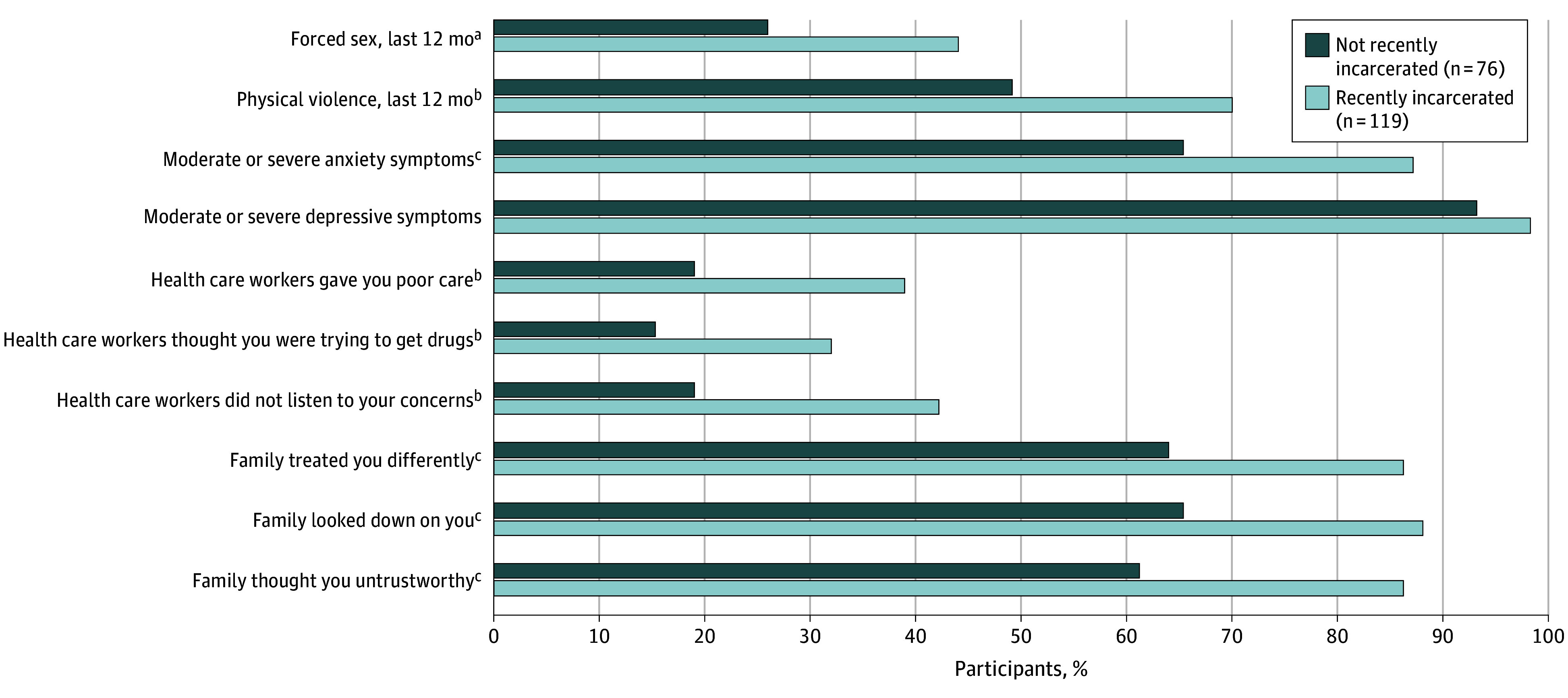
Stigma, Violence, and Mental Health Among Women Who Used Heroin in Tanzania, by Incarceration Status (N = 195) ^a^*P* < .05. ^b^*P* < .01. ^c^*P* < .001.

### Regression Results

In adjusted modified Poisson regression models (Table 2), recent incarceration was associated with higher sexual concurrency (101 of 119 [84.9%] vs 41 of 76 [54.0%]; aPR, 1.43; 95% CI, 1.16-1.78) and more than 9 times the prevalence of HIV care interruptions among 54 women who used heroin and were living with HIV (9 of 27 [33.3%] vs 1 of 24 [4.2%]; aPR, 9.74; 95% CI, 1.22-77.22). Incarceration was also associated with more than 5 times the prevalence of stimulant use in the past 6 months (26 of 119 [21.9%] vs 3 of 76 [4.0%]; aPR, 5.60; 95% CI, 1.63-19.28) and more than 60% higher prevalence of lifetime nonfatal overdose (51 of 119 [42.9%] vs 17 of 76 [22.4%]; aPR, 1.62; 95% CI, 1.01-2.61).

**Table 2.** Results From Modified Poisson Regression of HIV and Drug Use-Related Outcomes on Recent Incarceration Status Among Women Who Use Drugs in Tanzania (N = 195)^a^

Outcome	No./No. (%) [95% CI]		Regression results	
	Recently incarcerated	Not recently incarcerated	aPR (95% CI)	*P* value
HIV testing, past 6 mo	108/119 (90.8) [84.0-94.8]	68/76 (89.5) [80.1-94.7]	0.97 (0.88-1.07)	.51
Living with HIV	28/108 (25.9) [18.5-35.1]	26/68 (38.2) [27.3-50.5]	0.80 (0.50-1.28)	.35
Enrolled in HIV care^b^	27/28 (96.4) [77.0-99.5]	24/26 (92.3) [72.5-98.2]	1.04 (0.93-1.16)	.47
Stopped care, past 6 mo^c^	9/27 (33.3) [17.8-53.6]	1/24 (4.2) [0.5-26.5]	9.74 (1.22-77.22)	.03
Sexual concurrency, past mo	101/119 (84.9) [77.2-90.3]	41/76 (54.0) [42.5-64.9]	1.43 (1.16-1.78)	.001
Injection drug use, past 6 mo	14/119 (11.8) [7.1-19.0]	11/76 (14.5) [8.1-24.5]	0.73 (0.34-1.60)	.44
Stimulant use, past 6 mo	26/119 (21.9) [15.3-30.3]	3/76 (4.0) [1.3-11.7]	5.60 (1.63-19.28)	.006
Polysubstance use, past 6 mo	109/119 (91.6) [85.1-95.9]	62/76 (81.6) [71.0-89.5]	1.11 (0.99-1.25)	.09
Lifetime nonfatal overdose	51/119 (42.9) [34.2-52.0]	17/76 (22.4) [14.3-33.3]	1.62 (1.01-2.61)	.05

^a^ All analyses adjusted for age, education, homelessness in the past 6 months, and duration of heroin use in years.

^b^ Among women who used heroin living with HIV; n = 54.

^c^ Among women who used heroin living with HIV who reported being linked to care; n = 51.

## Discussion

To our knowledge, this was one of the first studies to characterize HIV and drug use-related outcomes among recently incarcerated women who used heroin in sub-Saharan Africa. We identified converging psychosocial and structural vulnerabilities and behavioral risks among women who were recently incarcerated, including higher exposure to violence, homelessness, sexual concurrency, and lifetime nonfatal overdose. Taken together, these may exacerbate HIV disparities already present among women who used heroin.^16,18,23^

We found incarceration was associated with higher rates of lifetime nonfatal overdose. Additional analyses from this study showed overdose was associated with attempting to stop heroin, suggesting potential heroin dependence in this sample.^20^ Indeed, for women who used heroin and experienced heroin dependence, incarceration may present a period of increased overdose risk given cessation of heroin use while confined. While research on incarceration and overdose in sub-Saharan Africa is limited, evidence from other settings has identified incarceration as a major risk factor for both fatal and nonfatal overdose.^4,5,6^ More research is needed in Tanzania and sub-Saharan Africa more broadly to understand the mechanisms through which incarceration may contribute to periods of overdose risk.

While we did not find associations between incarceration and HIV status, incarceration was associated with sexual concurrency. Sexual concurrency was reported by more than 50% of our sample regardless of incarceration history, potentially explained by high rates of sex work among women who used heroin as identified in our prior qualitative work with this population.^24^ While we did not directly measure current engagement in sex work, our measure of last-month income from sex can be used as a proxy for current engagement in sex work. Given that recently incarcerated women reported higher earnings from sex in the last month, they may have been more actively engaged in sex work than other women who used heroin in the study. Further research is warranted to disentangle sex work, drug use, and incarceration-related vulnerabilities among women who used heroin.

In our sample, reporting of concurrent stimulant use was markedly higher among recently incarcerated women who used heroin compared with other women who used heroin. While studies of stimulant use and HIV have tended to focus on cisgender men, emerging evidence shows stimulant use is associated with faster clinical HIV progression^7^ and AIDS-related mortality^8^ among cisgender women in the US and with increased HIV viremia among female sex workers in South Africa.^9^ Research and intervention for women who used heroin, including women who used heroin, should continue to prioritize the role of stimulants and polysubstance use among women.

While a small proportion of women who used heroin in our sample were living with HIV, HIV care stoppages in this group were much more common among those with incarceration history. This mirrors findings from other sub-Saharan Africa settings.^25,26^ Notably, while the literature often shows increased engagement in the HIV care continuum during periods of incarceration, in many settings this engagement declines during the postrelease period.^27,28^

These findings have important implications for practice and policy. First, given high criminalization of sex work and drug use in Tanzania and in sub-Saharan Africa more broadly,^29^ interventions addressing policing practices may address consequences associated with incarceration among women who used heroin. Given incarceration was associated with adverse outcomes among women who used heroin in our study (eg, stigma, overdose, and violence), we recommend the use of prearrest diversion programs for women accused of certain drug-related crimes (eg, possession and use of a controlled substance). Prearrest diversion to treatment programs may reduce crime and overdose fatalities among individuals with substance use disorders who encounter law enforcement.^30,31^ Tanzania’s Drug Control and Enforcement Act emphasized the right to health care for people who use drugs, including treatment as an alternative to imprisonment for cases in which addiction is deemed the cause of a criminal offense.^32^ However, limited access to legal services among people who used drugs,^33^ paired with widespread societal stigma toward people who used drugs,^34^ may limit the extent to which these options are offered to or pursued by people who used drugs.

Second, these findings could inform development and evaluation of interventions to reduce service interruptions and ensure appropriate provision of HIV and harm reduction services during incarceration—including expansion of onsite services. This could include strategies, such as voluntary screening for substance use disorder during incarceration and expansion of medications for opioid use disorder in carceral settings. In a collaborative manner, criminal legal and health care practitioners should connect women who used heroin with resources (eg, treatment and therapy) to address their health needs. For example, in the southern highlands of Tanzania, a community-led crisis response team has been established which partners with the Government of Tanzania and criminal legal practitioners (eg, prison officers, judicial officers, and law enforcement) to facilitate legal support and access to methadone for detainees who use drugs.^33^ Structural interventions, such as establishment of satellite methadone clinics in carceral settings with a large volume of people who use drugs, have also been effective at ensuring continuity of services for people who use drugs during periods of incarceration.^33^

Third, we recommend tailored strategies to promote HIV and harm reduction service linkage and support during reentry periods. While the cross-sectional nature of our study precluded us from assessing how behaviors changed during incarceration and reentry, elevated stimulant use, sexual concurrency, and HIV care stoppage occurred during the same 6-month window as incarceration, suggesting potential increases in these outcomes during reentry. Others have shown engagement in the HIV care cascade declines after release from carceral settings—often declining below levels prior to incarceration^35^—and patient navigation interventions and substance use treatment strategies show promise in improving postrelease outcomes.^36^ For formerly incarcerated people living with HIV in South Africa, community adherence clubs were found to be an acceptable differentiated care strategy to promote sustained HIV care engagement during the reentry period.^37^ Differentiated models of HIV care have also been found to be preferred among individuals released from prison in Zambia.^38^ Similar models should be explored to facilitate linkage to HIV and substance use services for people who use drugs in Tanzania.

Finally, given the high burden of mental health concerns and violence reported by recently incarcerated women who used heroin in our study, there is a need for psychosocial support services, including violence prevention and aftercare, for women who used heroin encountering Tanzania’s criminal legal system. Women who used heroin may be battling trauma and other life stressors that influence their substance use behavior.^39^ Service linkages for women who used heroin in carceral settings—including prearrest diversion to treatment—may support women in addressing life challenges. For example, cognitive behavioral therapy is a method shown to be effective in reducing substance-using behavior among women who used heroin.^40^

### Limitations

This study has limitations. First, this was an exploratory analysis conducted with a small sample of women who used heroin, and findings may not apply to women who used heroin in other settings. Additional research to understand incarceration experiences among women who used heroin and their impacts on future outcomes is warranted. Second, our reliance on cross-sectional data limits our ability to infer causal associations between incarceration and the outcomes assessed. Third, our use of self-reported data introduces potential information bias and misclassification of outcomes, which could bias observed associations toward the null. Additionally, some measures of socioeconomic status were limited, including income, which was only reported in the last 30 days and is likely to fluctuate for women who used heroin. Fourth, this was a secondary analysis, and the original study was not designed to assess incarceration. Therefore, we had a limited number of incarceration and arrest-related measures available in our dataset, precluding us from fully characterizing incarceration. Furthermore, we were unable to assess the timing of outcomes in relation to incarceration. For example, we were unable to assess overdose before vs after incarceration; similarly, while incarceration and HIV care stoppages were reported in the same 6-month window, we cannot determine their temporality. Further research, including longitudinal studies, should intentionally include metrics evaluating incarceration experiences in terms of duration, type of facility, or access to health care resources in carceral settings, as well as key outcomes occurring before, during, and after incarceration.

## Conclusions

To our knowledge, this is one of the first studies to describe substance use and HIV vulnerabilities among recently incarcerated women who used heroin in sub-Saharan Africa. We identified a critical need to address elevated violence and mental health issues, along with stimulant use and concurrent partnerships which may drive HIV risk during incarceration and reentry. Multilevel interventions, including cross-sectoral collaborations between public health, medicine, and the criminal legal system in Tanzania, can address these challenges and promote the health and human rights of women who used heroin.

## Notes

Supplement.
Data Sharing Statement
