# Supplementary material for: Recent Incarceration and HIV Risk Among Women Who Use Heroin
Source: JAMA Netw Open. 2025 Jan 13;8(1):e2454455. doi: 10.1001/jamanetworkopen.2024.54455 (PMC11731222; doi:10.1001/jamanetworkopen.2024.54455)
Supplement: Supplement. — Data Sharing Statement [file jamanetwopen-e2454455-s001.pdf]

Data Sharing Statement

Atkins. Recent Incarceration and HIV Risk Among Women Who Use Heroin. *JAMA Netw Open*. Published January 13, 2025. doi:10.1001/jamanetworkopen.2024.54455

Data
Data available: No
